# Mercury and Selenium Accumulation in the Tissues of Stranded Bottlenose Dolphins (*Tursiops truncatus*) in Northeast Florida, 2013–2021

**DOI:** 10.3390/ani14111571

**Published:** 2024-05-25

**Authors:** Gretchen K. Bielmyer-Fraser, Julia M. Courville, Ashlen Ward, Mckenna M. Hardie

**Affiliations:** Millar Wilson Laboratory, Jacksonville University, Jacksonville, FL 32211, USA; jcourvi1@jacksonville.edu (J.M.C.); ashlenward@aol.com (A.W.); mhardie2@jacksonville.edu (M.M.H.)

**Keywords:** cetacean welfare, contamination, mercury, selenium

## Abstract

**Simple Summary:**

Mercury is a persistent and toxic metal that can affect marine life. Bottlenose dolphins are particularly vulnerable to mercury accumulation and toxicity because they are top predators and often inhabit near-shore environments with elevated mercury concentrations. This research assessed mercury accumulation in the tissues of bottlenose dolphins that were stranded during two time periods, 2013–2015 (dolphins that were stranded at an unusually high rate; heavily infected with morbillivirus) and 2016–2021 (dolphins that were stranded at a normal rate). We hypothesized that the immunosuppressed individuals would have higher mercury concentrations. Tissue selenium accumulation was also measured, as selenium has been shown to reduce mercury toxicity. Differences in tissue mercury concentrations were not observed between the two time periods; however, the tissue mercury concentrations were higher in adults than juveniles. The selenium distribution in the bottlenose dolphins was significantly different between the two time periods. These results suggest that selenium may not have been as protective against mercury toxicity in the bottlenose dolphins that were stranded during the UME.

**Abstract:**

Bottlenose dolphins (*Tursiops truncatus*) are long-lived marine mammals, upper-level predators, and they inhabit near-shore environments, which increases their exposure to pollution. Mercury is a ubiquitous and persistent metal pollutant that can bioaccumulate and biomagnify up the food chain. Dolphins are known to accumulate mercury, and limited research has shown that mercury exposure can weaken the immune system of dolphins. The objectives of this study were to assess the mercury concentrations in the tissues (muscle, small intestine, liver) of stranded bottlenose dolphins and to compare the tissue mercury levels in dolphins that were stranded during the 2013–2015 morbillivirus Unusual Mortality Event (UME; immunosuppressed individuals) with the levels of those that were stranded at a normal rate (2016–2021). Selenium has been shown to reduce mercury toxicity in many animals; therefore, tissue selenium concentration and the molar ratio of selenium to mercury were also assessed. The tissue mercury (muscle, liver) and selenium (liver) concentrations increased with the age of the dolphins, with the liver accumulating the highest concentrations. No sex differences were observed in the mercury and selenium concentrations. While differences in tissue mercury concentrations were not observed due to the UME, the selenium accumulation profiles were significantly different between the two time periods. These results suggest that selenium may not have been as protective against mercury toxicity in the bottlenose dolphins that were stranded during the UME, possibly due to infection with morbillivirus.

## 1. Introduction

Mercury is a heavy metal commonly found in marine environments, primarily due to human activities [1,2,3]. Coal combustion accounts for most mercury emissions, followed by metal smelting, cement production, and, to a minor extent, waste incineration, the chlorine alkali industry, and the steel industry [3,4]. Mercury can also enter the marine environment through natural sources, notably in the Mediterranean. Mercury primarily enters aquatic systems in its inorganic form and can become methylated at the surface layer and in sediment via sulfate-reducing marine bacteria and carbon decomposition [5]. Mercury exposure in wildlife occurs mainly through the diet as methylmercury, which can biomagnify up the food chain [6,7,8]. The total amount of mercury accumulated varies based on factors such as size, age, sex, prey preference, and habitat use [8,9,10,11].

Bottlenose dolphins (*Tursiops truncatus*) are long-lived marine mammals inhabiting tropical and temperate coastal waters near anthropogenic pollution [6,11,12,13,14,15]. As top predators, dolphins serve as bioindicators of contaminants for multiple members of the ecosystem, including humans, whose diets overlap with those of dolphins (e.g., fish and cephalopods) [6,14,16,17,18].

Dolphins and other mammals can accumulate mercury [6,9,13,19,20,21,22], and mercury is known to cause neurotoxic effects [23]. Limited studies have shown that mercury can also weaken the immune system of bottlenose dolphins [11,14,24]). Cámara-Pellissó et al. [24] reported a significant reduction in the immune system response of bottlenose dolphins, with a decreased ability of the white blood cells to engulf foreign bodies and bacteria, after mercury exposure in vitro. White blood cell count decreased after exposure to 1 mg/L of mercury, and they were unable to destroy harmful cells after exposure to 5 mg/L of mercury [24]. Population health assessments conducted on bottlenose dolphins in the Indian River Lagoon in Florida from 2003 to 2008 [25] showed that dolphins with positive morbillivirus antibody titers had significantly reduced mitogen-induced T lymphocyte proliferation responses, as well as significant decreases in CD4+ lymphocytes. Reif et al. [25] reported associations between increased total mercury accumulation (the blood and skin) and both a decrease in white blood cells (lymphocytes, eosinophils, and platelets) and an increase in immune globulins in bottlenose dolphins. Further, monocyte phagocytic activity and plasma lysozyme concentration increased with increasing mercury in the blood [25]. 

The essential element selenium often accumulates with mercury [9] and can protect against mercury toxicity; however, the degree of protection is dependent on the species of selenium, the cell type, and the end points assessed [26,27,28]. Selenium replaces sulfur within the methylmercury–cysteine complex, forming an insoluble compound (HgSe) which reduces bioavailability and alleviates toxicity [29]. Molar ratios of selenium to mercury greater than 1:1 reduced mercury toxicity in mammalian studies [30]. Exposure to multiple stressors may lessen the protective effect of selenium. Manhães et al. [31] reported changes in the selenium body burden in Guiana dolphins infected with morbillivirus, suggesting that morbillivirus disabled the selenium-induced mercury detoxification mechanism in the liver. Specifically, selenium was reduced in a variety of tissues (e.g., the muscles and liver), and the total mercury was increased in the liver. The authors reported a liberation of methylmercury from the muscles in Guiana dolphins infected with morbillivirus [31]. Immunocompromised individuals could accumulate higher metal concentrations and/or have reduced detoxification mechanisms, thereby increasing their susceptibility to metal toxicity [31]. Exerting energy to combat a virus or bacteria would, theoretically, leave less energy for contaminant detoxification and excretion. 

A marine mammal stranding occurs when an animal is found dead (on the beach or floating in the water) or alive but is not a suitable candidate for release because it needs medical attention or is exhibiting abnormal behavior [32]. Strandings can result from natural and/or anthropogenic reasons [33]. A mass stranding event is when two or more animals (not including a mom and calf pair) are stranded in proximity to each other in time and space, usually occurring over the course of several hours to days and in one location or several locations [34]. An Unusual Mortality Event (UME) is an unexpected stranding which involves significant mortality within a marine mammal population and requires an immediate response (Marine Mammal Protection Act [MMPA] Title IV) [35]. The 2013 to 2015 cetacean morbillivirus UME resulted in 1614 bottlenose dolphins being stranded in nine states (New York to Florida) on the eastern Atlantic Coast of the United States from 1 July 2013 to 1 March 2015 [36]. The state of Florida had the second highest number of strandings, with 92% seroprevalence for morbillivirus [36]. Live dolphins with the disease exhibit clinical signs consisting of tremors, poor lipid reserves, poor nutritional state, and high burdens of ectoparasites and epibionts [37,38].

The objectives of this study were to assess the mercury and selenium concentrations in the tissues of juvenile and adult bottlenose dolphins and to the compare mercury and selenium body burdens between the dolphins that were stranded during the 2013–2015 morbillivirus UME (immunosuppressed individuals) and those that were stranded at a normal rate (2016–2021). We hypothesized that the mercury concentrations would be higher in the UME dolphins than the dolphins that were stranded at a normal rate because the presence of morbillivirus in the UME dolphins may have reduced the energy necessary for mercury detoxification and/or excretion. Secondly, we hypothesized that the relationship between selenium and mercury would be disrupted in the UME dolphins and the selenium levels would be lower in the liver and muscles of the UME dolphins. The results of this study have implications for bottlenose dolphin management strategies and necropsy procedures, as well as human health [34].

## 2. Materials and Methods

### 2.1. Field Sites

**Figure 1:**
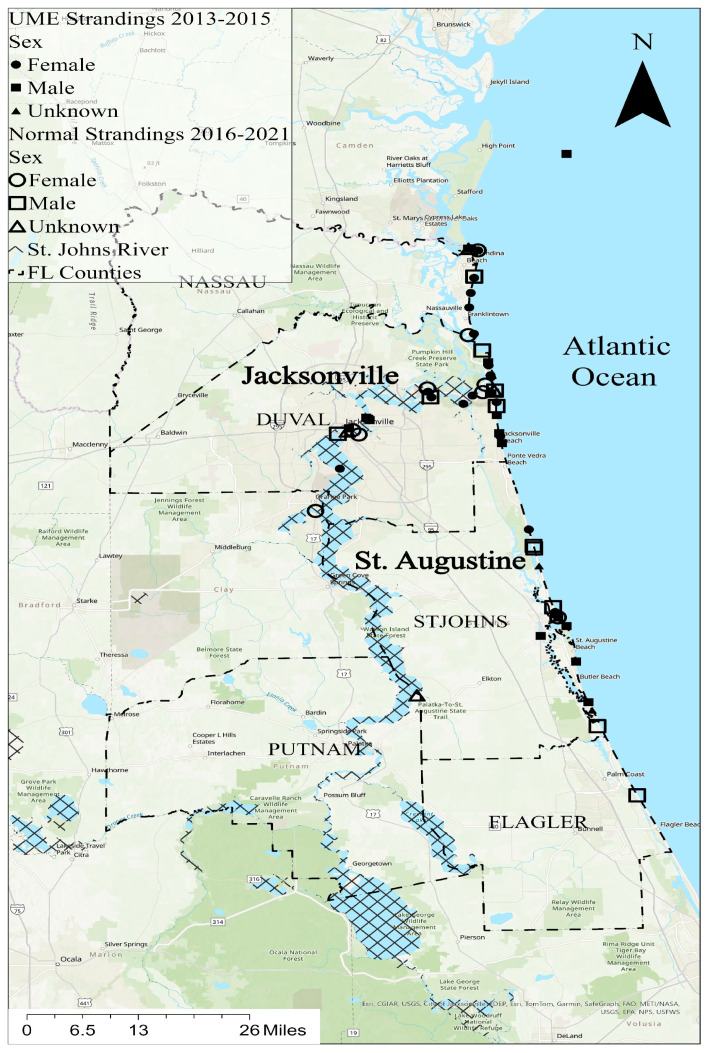
Stranding locations in northeast Florida; the bottlenose dolphins (*Tursiops truncatus*) used in this study are represented by symbols (circles = females, squares = males; triangles = unknown sex). Solid symbols indicate strandings during the 2013–2015 UME, and unfilled symbols indicate strandings at a normal rate from 2016 to 2021.

ArcGIS Pro 3.2 was used to make the GIS map showing the dolphin stranding locations (Figure 1). Two individuals (TtNEFL1613, unknown sex, and TtNEFL1385, female) had no location data and therefore were not included. The bottlenose dolphin strandings primarily occurred in northeast Florida, spanning six counties, including Putnam, Nassau, Duval, St. Johns, Flagler, and Clay (Figure 1). The majority of the strandings occurred in Duval County (*n* = 29) near Jacksonville, FL, and St. Johns County (*n* = 15) near St. Augustine, FL. A total of 42 dolphins were stranded along Florida’s northeast coast, and 14 were stranded in the St. Johns River, FL, predominantly near the mouth of the river. Mercury contamination is a known stressor in the St. Johns River [39].

Bottlenose dolphin carcasses were collected during two time periods, including the UME period (2013–2015) and the normal period (2016, 2019–2021). In the St. Johns River, 80% of the strandings were female during the normal period, while 44% of the strandings were female during the UME (Figure 1). Alternatively, females comprised 36% of the coastal strandings during the normal period and 52% of the coastal strandings during the UME.

### 2.2. Field Collection

Bottlenose dolphin tissue (muscle, small intestine, and liver) samples were collected from stranded carcasses by the Northeast Field laboratory from the Florida Fish and Wildlife Conservation Commission (FWC), and the samples were stored in labeled Whirl-Pak bags and placed in a cooler at the necropsy site. The samples used for this study were a subsample of those used for other studies; therefore, we were limited in the organ selection and the number of replicates. The samples were preserved in a −20 °C freezer at the Northeast Field laboratory and then transferred into a −80 °C freezer in the Marine Science building at Jacksonville University.

During the necropsies, the FWC identified the species (*Tursiops truncatus*) based on tooth count, identified sex, classified the decomposition on a scale from zero to five, and measured total length. Additionally, five teeth were collected from every carcass and analyzed to determine the age class of the animal. The biological information for each individual in this study is in Table 1. The tissue samples for this project were stored in individually labeled 2.0 mL cryovials in a −80 °C freezer. 

### 2.3. Sample Preparation

The tissue samples were thawed, and each sample was placed in a pre-weighed labeled empty aluminum weigh boat and massed to determine its wet weight (ww). The samples were then dried in an oven at 80 °C for 24 h, removed, allowed to cool to room temperature for at least twenty minutes, and then massed again to determine their dry weight (dw). The mean percent moisture values in the muscle, small intestine, and liver were 66.6, 51.9, and 64.8, respectively. The dried samples were crushed into powder using a porcelain mortar and pestle. Approximately 20 mg of the sample was then acidified using 100 µm of trace-metal-grade nitric acid (Thermo Fisher Scientific, Waltham, MA, USA) and heated in a water bath to 65 °C until complete digestion. Once it had liquefied completely, the tissue digest was diluted with ultrapure 18 mΩ Milli-Q^®^ water and vortexed. 

### 2.4. Mercury and Selenium Analysis

A DMA 80 automatic mercury analyzer (Milestone, Inc., Brondby, Denmark) was used to measure the total mercury concentration in the tissue samples, following EPA method 7473 [40]. A certified mercury stock solution (1000 mg Hg/L; Thermo Scientific) was used to make standard dilutions for instrument calibration. Instrument blanks and procedural blanks were used in all the analyses. Standard reference materials (at least six replicates of each) with certified mercury values, including DORM-4 (fish protein) and DOLT-5 (dogfish liver) from the National Research Council Canada (NRCC), were processed in the same way as the samples, and the values were within acceptable limits. The limit of detection for mercury was 0.7 µg/L. 

Selenium was quantified in the digested tissue samples using atomic absorption spectroscopy with graphite furnace detection, following EPA method 7010. Certified selenium standards (PerkinElmer, Shelton, CT, USA) and blanks were used in all the analyses. Certified reference materials (DORM-4, fish protein, and DOLT-5, dogfish liver) from the NRCC were processed in the same way as the samples, and the values were within acceptable limits. The limit of detection for selenium was 1.2 µg/L.

### 2.5. Data Analysis

SigmaPlot 15.0 software (Inpixon, Palo Alto, CA, USA) was used for all the data analyses. Data were tested for normality and equality of variance using the Shapiro-Wilk and Brown-Forsythe tests, respectively. A one-way ANOVA and a pairwise multiple comparison procedure (e.g., Dunn’s Method, Tukey’s test) were performed to determine statistical differences in the mercury and selenium concentrations among tissue type, age class, sex, and time periods (i.e., the UME versus the normal period). Pearson’s Product Moment Correlation was used to determine the correlation between the mercury and selenium accumulation in each of the tissues. Multiple linear regression (MLR; stepwise forward and backward) analysis was performed using tissue mercury concentration, tissue selenium concentration, and the molar ratio of selenium to mercury as the dependent variables. Stepwise MLR analysis included the independent variables of stranding location, stranding code, age class, sex, and time period (the UME or the normal stranding period). In addition to the listed independent variables, mercury tissue concentration was used as an independent variable with selenium tissue concentration as the dependent variable, and selenium tissue concentration was used as an independent variable with mercury tissue concentration as the dependent variable. A positive coefficient indicates that an increase in the independent (predictor) variable corresponds to an increase in the dependent variable.

## 3. Results

This study received tissue samples from a total of 64 individuals, including 28 females and 29 males (Table 1). There were 25 adults ranging from 208 to 298 cm in total length and 36 juveniles ranging from 100 to 254 cm in total length (Table 1). 

**Figure 2:**
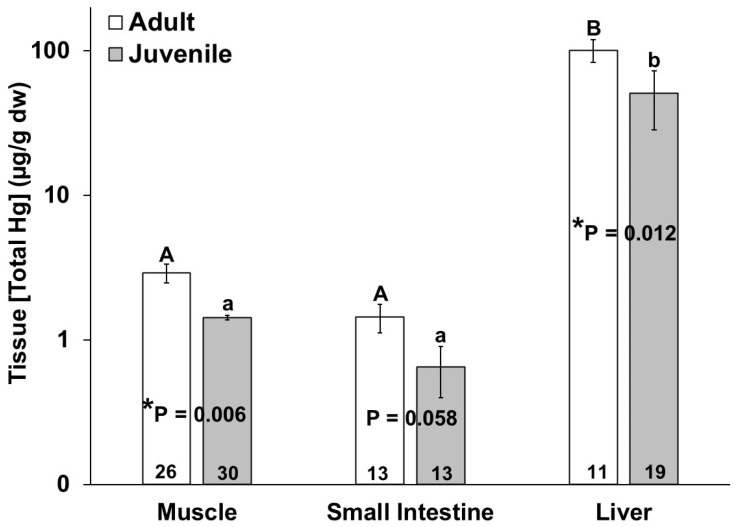
Total mercury (THg) concentration in muscle, small intestine, and liver of adult (white bars) and juvenile (gray bars) bottlenose dolphins (*Tursiops truncatus*) stranded in northeast Florida from 2013 to 2021. Different uppercase letters (adults) and lowercase letters (juveniles) represent significant differences (*p* ≤ 0.05) in mercury concentrations among tissues. Asterisks and *p*-values ≤ 0.05 indicate a significant difference in the mercury concentration in a particular tissue between adults and juveniles. The number at the base of each column represents sample size. Note the logarithmic scale on the Y-axis.

**Figure 3:**
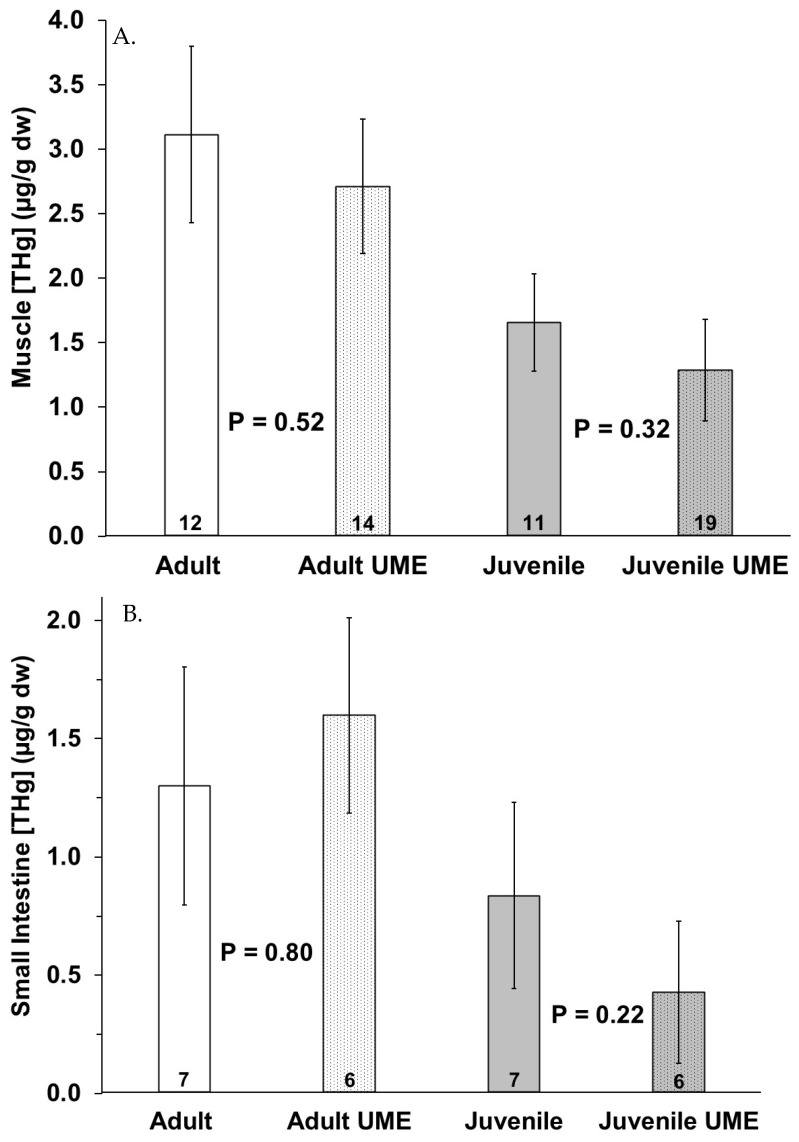

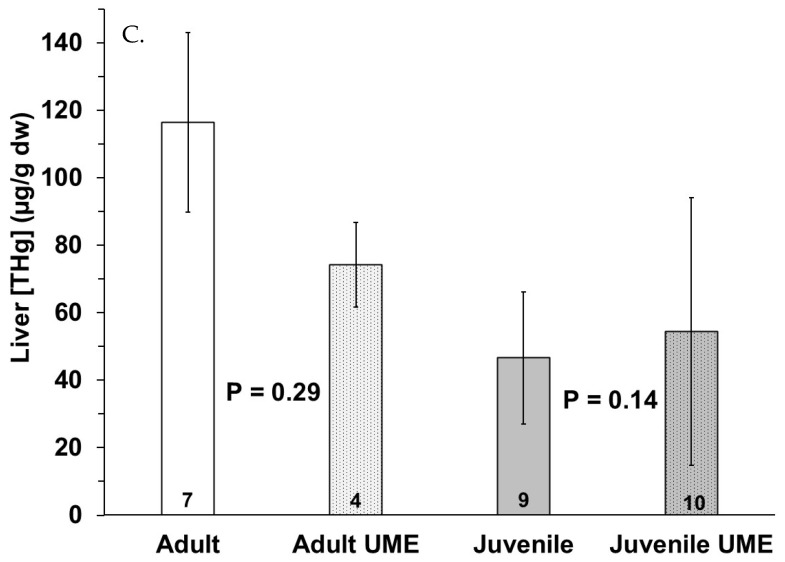
Total mercury (THg) in (**A**) muscle, (**B**) small intestine, and (**C**) liver of adult (white bars) and juvenile (gray bars) bottlenose dolphins (*Tursiops truncatus*) stranded in northeast Florida during the 2013–2015 UME (dotted bars) and during normal stranding years (2016–2021; solid bars). The number at the base of each column represents sample size. *p*-values ≤ 0.05 indicate a significant difference within an age class between the two stranding periods (normal and UME).

No significant differences in mercury or selenium tissue concentration were observed due to sex. The adult bottlenose dolphins had higher total mercury in their muscles (*p* = 0.011), livers (*p* = 0.018) and small intestines, for which this approached significance (*p* = 0.058), as compared to the juveniles (Figure 2). In comparing the bottlenose dolphins that were stranded during the UME, the adults had significantly higher intestinal mercury than the juveniles (*p* = 0.024). Additionally, the total mercury concentration in the liver was significantly higher than the total mercury in the muscle and small intestine for all individuals (Figure 2). The total mercury concentrations (µg/g dw) ranged from 0.08 to 9.53 in the muscles, from 0.04 to 4.17 in the small intestines, and from 0.26 to 398 in the livers of the bottlenose dolphins in this study (Figure 2). No significant differences in the tissue (muscle, small intestine, and liver) total mercury concentrations were observed in the adults or juveniles due to stranding period (UME versus normal; Figure 3). 

MLR analysis showed that age class was the only parameter that positively affected (*p* = 0.033) the muscle mercury concentration of the variables assessed. The following MLR equation shows this relationship (R^2^ = 0.286; adjusted R^2^ = 0.250).

Mercury concentration (nmol/g dw) = −3.655 + (9.804 × Age Class)

For the liver, only selenium concentration significantly influenced mercury concentration (*p* < 0.001); however, the power of the test was below the desirable value of 0.80. Therefore, a difference was less likely to be detected due to other variables when one existed. The following MLR equation shows this relationship (R^2^ = 0.621; adjusted R^2^ = 0.607).

Mercury concentration (nmol/g dw) = 159.520 + (0.178 × Selenium concentration (nmol/g dw))

No significant variables were seen to influence intestinal mercury concentration according to the MLR analysis.

**Figure 4:**
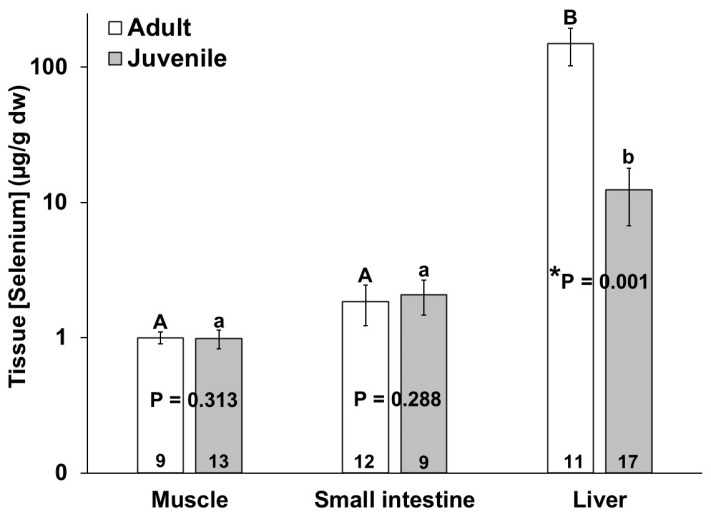
Selenium concentration in muscle, small intestine, and liver of adult (white bars) and juvenile (gray bars) bottlenose dolphins (*Tursiops truncatus*) stranded in northeast Florida from 2013 to 2021. Different uppercase letters (adults) and lowercase letters (juveniles) represent significant differences (*p* ≤ 0.05) in mercury concentrations among tissues. Asterisks and *p*-values ≤ 0.05 indicate significant differences in the mercury concentrations in a particular tissue between adults and juveniles. The number at the base of each column represents sample size. Note the logarithmic scale on the Y-axis.

The adult bottlenose dolphins also had a higher selenium concentration in their livers as compared to the juveniles; however, no significant differences in the muscle and small intestine selenium concentrations were detected between the age classes (Figure 4). Similar to mercury, the mean selenium concentration in all individuals was highest in the liver as compared to the muscle and small intestine (Figure 4). The selenium concentrations (µg/g dw) ranged from 0.1 to 1.5 in the muscles, from below detection to 5.7 in the small intestines, and from below detection to 448 in livers of the bottlenose dolphins in this study (Figure 4). 

Mercury accumulation and selenium accumulation in the livers of the bottlenose dolphins were significantly positively correlated (R^2^ = 0.463, *p* = 0.0076, *n* = 32), but no such correlation was found for the other two tissues.

**Figure 5:**
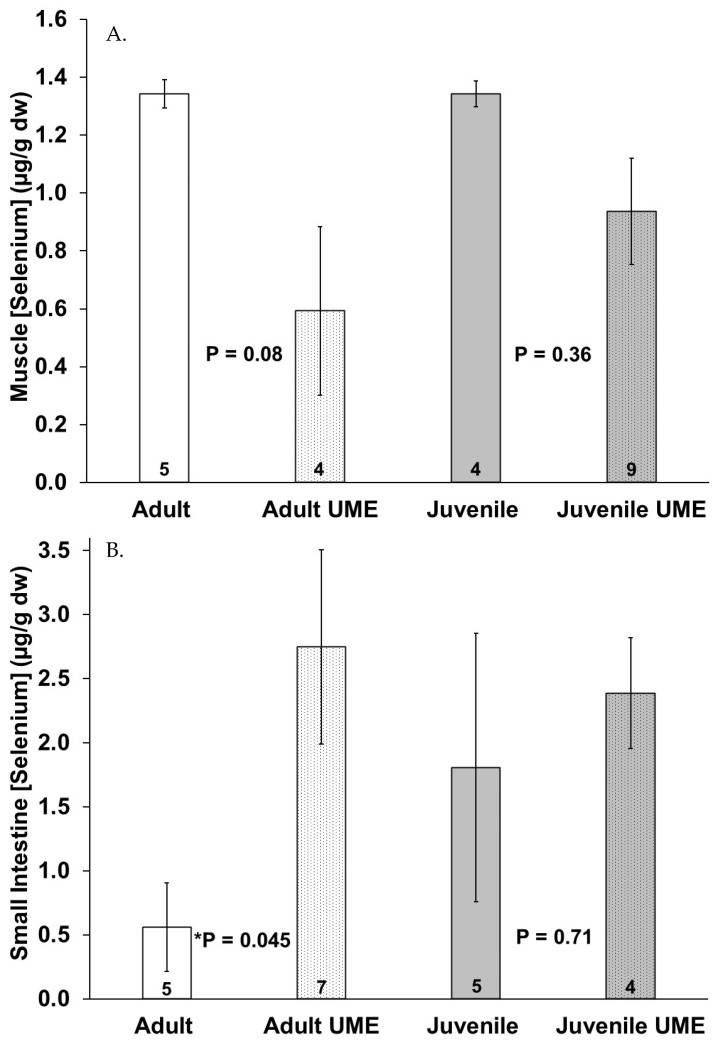

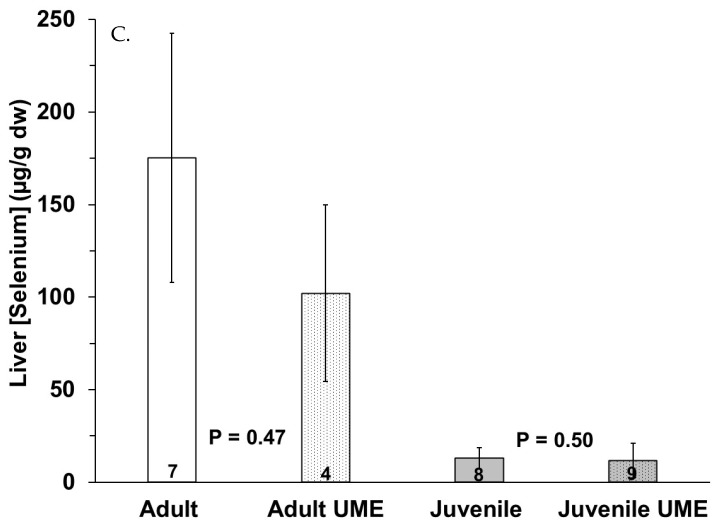
Selenium concentration in (**A**) muscle, (**B**) small intestine, and (**C**) liver of adult (white bars) and juvenile (gray bars) bottlenose dolphins (*Tursiops truncatus*) stranded in northeast Florida during the 2013–2015 UME (dotted bars) and during normal stranding years (2016–2021; solid bars). The number at the base of each column represents sample size. Asterisks and *p*-values ≤ 0.05 indicate a significant difference within an age class between the two stranding periods (normal and UME).

Figure 5 shows a trend of decreased selenium concentrations in the muscle and liver and increased selenium concentrations in the small intestine in the juveniles and especially the adults that were stranded during the UME as compared to the normal years. Adults that were stranded during the UME had significantly (*p* = 0.045) higher selenium levels in their intestines as compared to adults that were stranded during the normal years. When all the individuals that were stranded during the UME were compared to those that were stranded during the normal years, there were lower selenium levels in the muscles (*p* = 0.012) and higher selenium levels in the small intestine (*p* = 0.024) in the bottlenose dolphins that were stranded during the UME.

The MLR analysis showed that selenium concentration in the muscles was significantly (*p* = 0.012) affected by the stranding period (positively influenced in individuals stranded during the normal period), as shown by the following equation (R^2^ = 0.263; adjusted R^2^ = 0.228):

Selenium concentration (nmol/g dw) = 4.255 + (6.272 × Stranding Period).

No significant variables were seen to significantly affect intestinal selenium concentration according to the MLR analysis; however, the power of the test was below 0.80. Selenium concentration in the liver was significantly influenced individually by mercury concentration (*p* < 0.011; positive effect), age class (*p* = 0.011; positive effect), and stranding period (*p* = 0.011; positive effect of the normal stranding period). However, interactions were observed among the variables when performing the MLR analysis. The best MLR (R^2^ = 0.662; adjusted R^2^ = 0.619) included all three variables in the following equation, with only mercury concentration found to be significant (*p* < 0.001):

Selenium concentration (nmol/g dw) = −945.003 + (26.829 × Stranding Period) + (586.598 × Age Class) + (3.048 × Mercury concentration (nmol/g)).

After removing mercury concentration, the subsequent MLR detected a significant effect of age class (*p* = 0.005), although the predictive capabilities of the model decreased (R^2^ = 0.345; adjusted R^2^ = 0.293):

Selenium concentration (nmol/g dw) = −1909.228 + (346.518 × Stranding Period) + (1594.769 × Age Class).

Lastly, in removing both mercury concentration and age class, the MLR had very low predictive capability for the selenium concentration in the liver (R^2^ = 0.0934; adjusted R^2^ = 0.0598):

Selenium concentration (nmol/g dw) = −553.975 + (838.388 × Stranding Period).

Table 2 shows a trend of a decreased mean molar ratio of selenium:mercury in the muscle and liver and an increased ratio in the small intestine in the adult bottlenose dolphins that were stranded during the UME as compared to those that were stranded during the normal period. However, no significant differences were detected due to stranding period. Juveniles had significantly higher selenium:mercury ratios in their muscles and lower ratios in their livers as compared to adults. Further, the mean selenium:mercury molar ratio was below one in the muscles of the UME adults and the livers of all juveniles (Table 2).

The MLR analysis showed that the selenium:mercury molar ratios in the muscle were significantly affected by age class (*p* = 0.018), and the best MLR used both age class and stranding period as independent variables, as shown in the following equation (R^2^ = 0.320; adjusted R^2^ = 0.245):

Selenium:Mercury molar ratio = 2.983 − (1.716 × Age Class) + (1.091 × Stranding Period).

The selenium:mercury molar ratios in the small intestine were most affected by stranding period (*p* = 0.056) when both stranding period and age class were used as independent variables, with the following equation (R^2^ = 0.235; adjusted R^2^ = 0.139):

Selenium:Mercury molar ratio = 12.528 − (4.482 × Stranding Period) − (1.063 × Age Class).

When Age Class was removed the *p*-value for the influence of Stranding Period on selenium: mercury molar ratio in the small intestine increased to *p* = 0.063. The power of this test was below the desired power of 0.80. The selenium: mercury molar ratio in the liver was significantly affected by Age Class (*p* < 0.001) and Stranding Period (*p* = 0.044) individually, however, when both parameters were included as independent variables only Age Class (*p* = 0.001) was signficant. The best MLR included both parameters as shown by the following equation (R^2^ = 0.445; adjusted R^2^ = 0.399):

Selenium:Mercury molar ratio = −2.469 + (0.704 × Stranding Period) + (2.233 × Age Class).

## 4. Discussion

The mercury and selenium tissue concentrations in the bottlenose dolphins from this study are within the ranges of those reported for other cetaceans [6,9,18,41,42]. For example, García-Alvarez et al. [43] reported similar mercury (223.8 mg/kg dw) and selenium (68.63 mg/kg dw) concentrations in the liver tissue of bottlenose dolphins from near the Canary Islands to those reported in the present study. Durden et al. [9] reported mean mercury and selenium concentrations of 5.68 (0.26–47) mg/kg ww and 1.92 (0.75–16.1) mg/kg ww in the muscle and 73.0 mg/kg ww (0.42–240) and 29.8 (1.20–90.7) mg/kg ww in the liver of stranded bottlenose dolphins from the Indian River Lagoon, FL. Similarly, in the present study, using percent moisture conversion factors, the mean mercury and selenium values were 0.70 and 0.30 mg/kg ww in the muscle, 0.67 and 0.90 mg/kg ww in the small intestine, and 38.9 and 22.7 mg/kg ww in the liver, respectively. Likewise, Guiana dolphins had similar mercury concentrations in their muscle (1.07 mg/kg ww; [21]) and liver (0.53–132 mg/kg; ww; [18]) tissue as in the present study. Guiana dolphins also had liver tissue selenium concentrations (0.17–74.8 mg/kg ww; [18]) within the range reported here. While Squadrone et al. [44] reported similar mercury and selenium concentrations in the muscles and livers of sperm whales (*Physeter macrocephalus*) to those in the present study, Cáceres-Saez et al. [45] reported higher concentrations of both elements in the muscles and livers of false killer whales (*Pseudorca crassidens*). Lower values of mercury and selenium were reported in the smaller coastal South American dolphin (*Pontoporia blainvillei*; [10,46]) than the bottlenose dolphins in this study, possibly reflecting differences in body mass and the propensity for mercury to biomagnify. Sedak et al. [47] noted higher mercury levels in Risso’s dolphins as compared to striped dolphins, attributing the difference in mercury levels to the larger size of Risso’s dolphins.

In mammals, the liver is the main detoxification organ for a variety of contaminants, including mercury [13,23,48,49,50]. It follows that the mercury concentrations were highest in the liver, as compared to the other organs measured in the present study (i.e., small intestine, muscle), which is consistent with the scientific literature [23,47,48,51,52]. Wagemann and Muir [53] suggested that mercury toxicity can occur when mercury concentrations exceed a threshold value of 100 mg/kg ww in the livers of marine mammals, whereas Rawson et al. [54] reported liver damage and significant health effects in dolphins with liver mercury concentrations exceeding 60 mg/kg ww. In this study, there were eight individuals with mercury concentrations exceeding 100 mg/kg ww in their livers, with nine additional individuals with liver mercury concentrations exceeding 60 mg/kg ww, possibly contributing to their morbidity. The total mercury concentrations in the small intestine of bottlenose dolphins are not commonly reported. In this study, the mercury concentrations in the small intestine were comparable to the muscle mercury levels.

Durden et al. [9] reported a positive correlation between tissue (muscle and liver) mercury concentrations and the age of bottlenose dolphins, like other studies [43,47]. In the present study, we found significant differences in the mercury concentration in the muscle and liver between age classes, with a positive effect of age class on the mercury concentration in the muscle. We also found a positive effect of age class on the selenium concentration in the liver; however, no correlations were observed between age and selenium in the muscle or small intestine. These observations are consistent with another bottlenose dolphin study [55]. The magnitude and length of exposure, animal diet, and longevity may also influence differences in element accumulation.

Selenium is an important trace element, essential for many biological functions, including metabolic, antioxidant, and reproductive activities [23,27,28]. Although not well understood, selenium has been shown to protect against mercury toxicity by binding and demethylating mercury [23]. Like the findings in this study, several others have noted a positive correlation between mercury accumulation and selenium accumulation in the livers of organisms, further supporting selenium’s role in detoxification [9,56,57,58]. Marumoto et al. [58] reported the co-localization of mercury and selenium in the livers of Indo-Pacific bottlenose dolphins. In addition to the liver, Durden et al. [9] reported a positive correlation between mercury and selenium levels in the muscle tissue, contrary to the findings of the present study.

In certain tissues (e.g., the liver, brain, kidneys, muscles), molar ratios of selenium to mercury greater than 1:1 may exert protection against mercury toxicity. Durden et al. [9] reported mean molar ratios of mercury to selenium of 1.03 in the liver and 1.17 in the muscle of bottlenose dolphins from the Indian River Lagoon, FL, similar to the calculated mean mercury-to-selenium ratios of 0.80 in the liver and 1.20 in the muscle of the bottlenose dolphins in the present study. Juveniles had selenium-to-mercury ratios less than one in their liver, regardless of the time of stranding, possibly increasing their susceptibility to mercury toxicity. In the adult bottlenose dolphins, the mean molar ratio of selenium to mercury was greater than one in the muscle and liver, suggesting some protection against mercury toxicity. The selenium-to-mercury ratios in the muscle were less than one, and the ratios increased (near significance; *p* = 0.07) in the small intestine in those adults that were stranded during the UME.

The effect of stranding period on selenium accumulation in the intestine is a novel finding. This increased selenium accumulation in the dolphins that were stranded during the UME could be due to a decrease in the absorption of or an increase in the excretion of selenium in the immunocompromised individuals. The redistribution of selenium, with lower concentrations in the muscle and liver, could remobilize more toxic forms of mercury [31]. Changes in diet and appetite may have also affected the observed values. Our results in bottlenose dolphins corroborate the observations (e.g., changes in tissue mercury and selenium distribution) that have been described in morbillivirus-infected Guiana dolphins [31]. The authors suggested that morbillivirus prevented selenium-induced mercury detoxification in the liver, but the process of this phenomenon is unknown. The authors also reported muscle loss, lower lipid percentages, and empty stomachs in Guiana dolphins that were stranded during the UME [31], all of which could have decreased their necessary selenium stores and lessened their ability to detoxify contaminants. More investigations are needed.

The occurrence of mass mortality events throughout the state of Florida and elsewhere has increased concerns about their potential causes, including contaminant exposure and the presence of multiple stressors in aquatic systems [9]. Mercury contamination is well documented in the Lower St. Johns River, where many of the bottlenose dolphins in this study were stranded [39]. The mercury concentrations in the sediments of the St. Johns River, FL, are well above the threshold effect concentration (freshwater) of 0.17 mg/kg and the threshold effect level (marine) of 0.13 mg/kg, with its mercury values reaching up to 0.70 mg/kg in recent years [59]. However, no effect of stranding site was found in this study.

Strandings are important to study for many reasons. The increased availability of quality data can be used to inform conservation and management strategies [34]. Stranding data allow policymakers to evaluate the status of marine mammals to determine whether a population should be designated as depleted and whether conservation plans are needed [60]. Studying strandings allows researchers to better understand the health and environment of marine mammals and evaluate and monitor human activities that might affect marine species [60]. The state of the animal that is stranded is an important consideration and limitation when using data derived from the examination of stranded animals. For example, many animals that are stranded may be malnourished, have infectious or degenerative diseases, or implicate other factors that may not be present in a healthy individual or population.

When UMEs are investigated, additional tissues and organs are examined and tested by researchers compared to during normal stranding events. Metals are not normally measured during the dolphin necropsies undertaken by the Florida Fish and Wildlife Conservation Commission (FWC) or during NOAA’s investigations. More research is needed to elucidate the interactions between mercury and selenium and to determine the levels of these elements that can exert toxic effects, particularly in combination with other stressors, like morbillivirus. During UMEs, it would be worthwhile to measure a suite of contaminants (notably total mercury and methylmercury) in multiple tissues (e.g., muscle and liver) to better assess the cause of death. The results from this study could help provide a better understanding of multiple stressors that can cause dolphin strandings and UMEs and may augment the necropsy procedures during these events.

## 5. Conclusions

In this study, changes in selenium body burden were observed in bottlenose dolphins that stranded during the 2013–2015 morbillivirus UME (immunosuppressed individuals) as compared with those that were stranded at a normal rate (2016–2021). The redistribution of selenium in these dolphins could have reduced selenium-induced protective effects against mercury toxicity, possibly resulting in increased concentrations of methylmercury. The tissue mercury concentrations were higher in the adults than the juveniles, and the liver accumulated the highest concentrations of both mercury and selenium. These results suggest that selenium may not be as protective against mercury toxicity in bottlenose dolphins, particularly adults, infected with morbillivirus, with the caveat that other factors independent of immune status may also play a role. Assessing the effects of multiple stressors, particularly in field situations, is complicated. More research is needed on this topic. UMEs are important to investigate to better understand the health of marine mammal populations and the health of the ocean and to give insight into larger environmental issues which may have implications for humans. This study provides new data about the influence of multiple stressors on this sentinel species and has implications for bottlenose dolphin management strategies and necropsy procedures.

## Figures and Tables

**Table 1:** Biological information for stranded bottlenose dolphins (*Tursiops truncatus*) which were used in this study from during a UME (2013–2015) and normal years (2016–2021). The stranding dates, locations, and decomposition codes (Dec Code) are also provided.

Date	Sample ID	UME	Sex	Age Class	Total Length (cm)	Tissue	Element	Dec Code	Location
3/21/2013	TtNEFL1314	N	M	Juvenile	188	L, M, SI L, M, SI	Se Hg	3	Coastal
21/11/2013	TtNEFL1345	Y	M	Juvenile	216	M, SI M, SI	Se Hg	3	Coastal
21/11/2013	TtNEFL1346	Y	M	Juvenile	105	M	Hg	3	Coastal
21/11/2013	TtNEFL1347	Y	M	Juvenile	136	L, M	Hg	3	Coastal
22/11/2013	TtNEFL1348	Y	F	Juvenile	120	M	Hg	3	Coastal
24/11/2013	TtNEFL1354	Y	M	Adult	257	M	Hg	3	Coastal
24/11/2013	TtNEFL1356	Y	UK	Juvenile	110	M	Hg	3	Coastal
26/11/2013	TtNEFL1361	Y	F	Juvenile	227	M	Hg	2	Coastal
27/11/2013	TtNEFL1362	Y	F	Adult	252	M	Hg	2	Coastal
28/11/2013	TtNEFL1363	Y	F	Juvenile	212	M	Hg	3	Coastal
1/12/2013	TtNEFL1366	Y	F	Adult	249	M	Hg	3	Coastal
6/12/2013	TtNEFL1373	Y	F	Juvenile	233	SI SI	Se Hg	3	Coastal
8/12/2013	TtNEFL1376	Y	F	Adult	277	M	Hg	3	Coastal
19/12/2013	TtNEFL1384	Y	F	Juvenile	214	M	Hg	3	Coastal
22/12/2013	TtNEFL1385	Y	F	Juvenile	UK	L, SI L, M	Se Hg	UK	Intra-coastal
27/12/2013	TtNEFL1387	Y	M	Juvenile	148	M	Hg	3	Coastal
24/3/2014	TtNEFL1416	Y	M	Juvenile	143	L, M	Hg	3	Coastal
24/3/2014	TtNEFL1417	Y	M	Juvenile	100	M	Hg	3	Coastal
25/3/2014	TtNEFL1418	Y	F	Juvenile	228	L	Hg	1,2	Coastal
4/4/2014	TtNEFL1419	Y	UK	UK	253	L, M, SI	Hg	3	Coastal
11/4/2014	TtNEFL1420	Y	F	Juvenile	197	M	Hg	2	Coastal
28/4/2014	TtNEFL1423	Y	M	Adult	298	L, M	Hg	3	Coastal
25/5/2014	TtNEFL1426	Y	F	Juvenile	185	L, SI L, M, SI	Se Hg	3	SJR
2/6/2014	TtNEFL1428	Y	F	Juvenile	217	L, M	Hg	3	SJR
9/6/2014	TtNEFL1429	Y	F	Adult	241	SI M	Se Hg	2	Coastal
17/6/2014	TtNEFL1430	Y	F	Adult	238	SI M, SI	Se Hg	2	Coastal
296/2014	TtNEFL1432	Y	M	Juvenile	198	L	Hg	3	Coastal
2/7/2014	TtNEFL1433	Y	M	Adult	258	L, SI M, SI	Se Hg	3	Coastal
7/7/2014	TtNEFL1435	Y	M	Juvenile	240	M	Hg	3	SJR
22/7/2014	TtNEFL1440	Y	F	Adult	237	L	Hg	3	SJR
29/7/2014	TtNEFL1444	Y	F	Juvenile	129	L, SI L, M, SI	Se Hg	3	SJR
7/8/2014	TtNEFL1445	Y	M	Adult	254	M	Hg	2	Coastal
9/8/2014	TtNEFL1446	Y	M	Adult	258	M	Hg	3	Coastal
19/8/2014	TtNEFL1448	Y	M	Juvenile	148	L, M, SI	Hg	3	Coastal
22/8/2014	TtNEFL1450	Y	M	Adult	247	L, SI L, M, SI	Se Hg	3	Coastal
29/8/2014	TtNEFL1453	Y	F	Juvenile	113	M	Hg	3	Coastal
3/9/2014	TtNEFL1454	Y	M	Adult	267	L, SI L, M, SI	Se Hg	3	Coastal
9/10/2014	TtNEFL1458	Y	M	Juvenile	242	L, M	Hg	3	SJR
24/11/2014	TtNEFL1462	Y	M	Adult	255	SI M, SI	Se Hg	3	Coastal
5/2/2015	TtNEFL1503	Y	F	Adult	255	M, SI M, SI	Se Hg	2	Coastal
1/3/2015	TtNEFL1506	N	UK	Adult	248	M	Hg	4	Coastal
5/3/2015	TtNEFL1507	N	M	Juvenile	183	SI M, SI	Se Hg	3	Coastal
29/3/2015	TtNEFL1510	N	F	Adult	269	M, SI	Hg	3	SJR
6/1/2016	TtNEFL1601	N	M	Juvenile	174	L	Hg	3	Coastal
19/2/2016	TtNEFL1602	N	M	Juvenile	117	M	Hg	3	Coastal
7/4/2016	TtNEFL1607	N	M	Juvenile	200	L, M, SI	Hg	3	MR
1/6/2016	TtNEFL1608	N	M	Adult	261	L, SI L, M, SI	Se Hg	3	Coastal
6/6/2016	TtNEFL1610	N	F	Adult	253	M	Hg	3	SJR
24/6/2016	TtNEFL1613	N	UK	UK	253	M	Hg	UK	UK
18/6/2016	TtNEFL1616	N	F	Juvenile	111	L	Hg	3	Coastal
25/8/2016	TtNEFL1621	N	F	Juvenile	101	SI SI	Se Hg	3	Coastal
30/8/2016	TtNEFL1624	N	M	Juvenile	212	L, M	Hg	3	Coastal
12/9/2016	TtNEFL1626	N	UK	Juvenile	218	M	Hg	4	Coastal
27/12/2016	TtNEFL1630	N	M	Juvenile	186	M	Hg	3	SJR
15/2/2019	TtNEFL1904	N	F	Adult	247	L, SI L, SI	Se Hg	3	SJR
21/6/2019	TtNEFL1914	N	F	Adult	247	L, M, SI L, M, SI	Se Hg	3	SJR
24/7/2019	TtNEFL1916	N	F	Juvenile	170	L, M, SI L, M, SI	Se Hg	3	SJR
6/8/2019	TtNEFL1918	N	M	Adult	271	L, M, SI L, M, SI	Se Hg	3	SJR
12/10/2019	TtNEFL1922	N	UK	Adult	208	L, M	Hg	4	SJR
2/12/2019	TtNEFL1923	N	F	Juvenile	162	L, M, SI	Hg	3	Nassau Sound
7/2/2020	TtNEFL2001	N	M	Juvenile	210	L, M, SI L, M, SI	Se Hg	2	Coastal
20/10/2020	TtNEFL2013	N	UK	UK	UK	L, M	Hg	4	SJR
8/4/2021	TtNEFL2109	N	M	Adult	267	L, M, SI L, M, SI	Se Hg	2	Coastal
3/5/2021	TtNEFL2112	N	F	Adult	UK	L, M, SI L, M, SI	Se Hg	2	SJR

UK = unknown; SI = small intestine; M = muscle; L = liver; SJR = St. Johns River; MR = Mantanzas River.

**Table 2:** Mean molar ratio of selenium to mercury (Se:Hg) in muscle, small intestine (SI), and liver tissue of adult and juvenile bottlenose dolphins (*Tursiops truncatus*) stranded during a UME (2013–2015) and normal years (2016–2021). Asterisks and *p*-values ≤ 0.05 indicate significant differences within an age class between the two stranding periods (normal and UME).

Age Class	Muscle Mean Molar Se:Hg	SI Mean Molar Se:Hg	Liver Mean Molar Se:Hg
Adult Normal	1.48 (±0.87)	0.97 (±1.63)	3.70 (±2.16)
Adult UME	0.75 (±1.02) *p* = 0.11	6.36 (±5.20) *p* = 0.07	1.67 (±1.73) *p* = 0.17
Juvenile Normal	3.62 (±2.18)	3.21 (±4.52)	0.91 (±0.95)
Juvenile UME	2.68 (±1.79) *p* = 0.44	6.80 (±5.66) *p* = 0.48	0.95 (±0.95) *p* = 0.63
Adult vs. Juvenile	* *p* = 0.01	*p* = 0.61	* *p* < 0.001

## Data Availability

The original contributions presented in the study are included in the article. Further inquiries can be directed to the corresponding author/s.

## References

[1] Knap A.H., Cook S.B., Cook C.B., Simmons J.A., Jones R.J., Murray A.E. (1991). Marine Environmental Studies to Determine the Impact of the Mass Burn Incinerator Proposed for Tynes Bay, Bermuda.

[2] Guzman H.M., Garcia E.M. (2002). Mercury levels in coral reefs along the Caribbean coast of Central America. Mar. Pollut. Bull..

[3] Teng H., Altaf A.R. (2022). Elemental mercury (Hg^0^) emission, hazards, and control: A brief review. J. Hazard. Mater..

[4] Yang W., Wang Z., Liu Y. (2020). Review on magnetic adsorbents for removal of elemental mercury from flue gas. Energ. Fuel.

[5] Mason R.P., Choi A.L., Fitzgerald W.F., Hammerschmidt C.R., Lamborg C.H., Soerensen A.L., Sunderland E.M. (2012). Mercury biogeochemical cycling in the ocean and policy implications. Environ. Res..

[6] Pompe-Gotal J., Srebocan E., Gomercic H., Prevendar Crnic A. (2010). Mercury concentrations in the tissues of bottlenose dolphins (*Tursiops truncatus*) and striped dolphins (*Stenella coeruloalba*) stranded on the Croatian Adriatic coast. Vet. Med..

[7] Wang S.L., Xu X.R., Sun Y.X., Liu J.L., Li H.B. (2013). Heavy metal pollution in coastal areas of South China: A review. Mar. Pollut. Bull..

[8] McMeans B.C., Arts M.T., Fisk A.T. (2015). Impacts of food web structure and feeding behavior on mercury exposure in Greenland sharks (*Somniosus microcephalus*). Sci. Total Environ..

[9] Durden W.N., Stolen M.K., Adams D.H., Stolen E.D. (2007). Mercury and selenium concentrations in stranded bottlenose dolphins from the Indian River Lagoon system, Florida. Bull. Mar. Sci..

[10] Seixas T.G., Kehrig H.A., Costa M., Fillmann G., Di Beneditto A.P.M., Secchi E.R., Moreira I. (2008). Total mercury, organic mercury and selenium in liver and kidney of a South American coastal dolphin. Environ. Pollut..

[11] Stavros H.W., Stolen M., Durden W.N., McFee W., Bossart G.D., Fair P.A. (2011). Correlation and toxicological inference of trace elements in tissues from stranded and free-ranging bottlenose dolphins (*Tursiops truncatus*). Chemosphere.

[12] Klinowska M. (1991). Dolphins, Porpoises and Whales of the World: The IUCN Red Data Book.

[13] Frodello J.P., Romeo M., Viale D. (2000). Distribution of mercury in the organs and tissues of five toothed-whale species of the Mediterranean. Environ. Pollut..

[14] Bilandžić N., Sedak M., Doki M., Gomercic M.D., Gomercic T., Zadravec M., Benic M., Crnic A.P. (2012). Toxic element concentrations in the bottlenose (*Tursiops truncatus*), Striped (*Stenella coeruleoalba*) and Risso’s (*Grampus griseus*) dolphins stranded in Eastern Adriatic Sea. Bull. Environ. Contam. Toxicol..

[15] Caldwell M. (2016). Historical evidence of *Tursiops truncatus* exhibiting habitat preference and seasonal fidelity in northeast Florida. Aquat. Mamm..

[16] Aguilar A., Borrel A., Pastor T. (1999). Biological factors affecting variability of persistent pollutant levels in cetaceans. J. Cetac. Res. Manag..

[17] Bossart G.D. (2006). Marine mammals as sentinel species for oceans and human health. Oceanography.

[18] Lailson-Brito J., Cruz R., Dorneles P.R., Andrade L., Azevedo Ade F., Fragoso A.B., Vidal L.G., Costa M.B., Bisi T.L., Almeida R. (2012). Mercury-selenium relationships in liver of Guiana dolphin: The possible role of Kupffer cells in the detoxification process by tiemannite formation. PLoS ONE.

[19] O’Shea T.J., Reynolds J.M., Rommel S.A. (1999). Environmental Contaminants and Marine Mammals. Biology of Marine Mammals.

[20] Ruelas-Inzunza J.R., Horvat M., Perez-Cortes H., Paez-Osuna F. (2003). Methylmercury and total mercury distribution in tissues of gray whales (*Eschrichtius robustus*) and spinner dolphins (*Stenella longirostris*) stranded along the lower Gulf of California, Mexico. Ciencias Marinas.

[21] De Moura J.F., Hacon Sde S., Vega C.M., Hauser-Davis R.A., de Campos R.C., Siciliano S. (2012). Guiana dolphins (*Sotalia guianensis*, Van Benédén 1864) as indicators of the bioaccumulation of total mercury along the coast of Rio de Janeiro state, Southeastern Brazil. Bull. Environ. Contam. Toxicol..

[22] Damseaux F., Kiszka J.J., Heithaus M.R., Scholl G., Eppe G., Thomé J.P., Lewis J., Hao W., Fontaine M.C., Das K. (2017). Spatial variation in the accumulation of POPs and mercury in bottlenose dolphins of the Lower Florida Keys and the coastal Everglades (South Florida). Environ. Pollut..

[23] Lopez-Berenguer G., Peñalver J., Martínez-López E. (2020). A critical review about neurotoxic effects in marine mammals of mercury and other trace elements. Chemosphere.

[24] Cámara Pellissó S., Muñoz M.J., Carballo M., Sánchez-Vizcaíno J.M. (2008). Determination of the immunotoxic potential of heavy metals on the functional activity of bottlenose dolphin leukocytes in vitro. Vet. Immunol. Immunopathol..

[25] Reif J., Schaefer A., Bossart G. (2015). Atlantic bottlenose dolphins (*Tursiops truncatus*) as a sentinel for exposure to mercury in humans: Closing the loop. Vet. Sci..

[26] Koeman J.H., Peeters W.H.M., Koudstaal-Hol C.H.M., Tjioe P.S., De Goeij J.J.M. (1973). Mercury-selenium correlations in marine mammals. Nature.

[27] Ralston N.V.C., Unrine J., Wallschlager D. (2008). Biogeochemistry and Analysis of Selenium and Its Species.

[28] Das K., Dupont A., De Pauw-Gillet M.C., Debier C., Siebert U. (2016). Absence of selenium protection against methylmercury toxicity in harbour seal leucocytes in vitro. Mar. Pollut. Bull..

[29] Zhang H., Feng X., Chan H.M., Larssen T. (2014). New insights into traditional health risk assessments of mercury exposure: Implications of selenium. Environ. Sci. Technol..

[30] Decataldo A., Di Leo A., Giandomencio S., Cardellicchio N. (2004). Association of metals (mercury, cadmium and zinc) with methallothionein-like proteins in storage organs of stranded dolphins from the Mediterranean Sea (Southern Italy). J. Environ. Monit..

[31] Manhães B.M.R., Santos-Neto E.B., Tovar L.R., Guari E.B., Flach L., Kasper D., Galvão P.M.A., Malm O., Gonçalves R.A., Bisi T.L. (2021). Changes in mercury distribution and its body burden in delphinids affected by a morbillivirus infection: Evidences of methylmercury intoxication in Guiana dolphin. Chemosphere.

[32] Gales N., Woods R., Vogelnest L., Woods R., Vogelnest L. (2008). Marine mammal strandings and the role of the veterinarian. Medicine of Australian Mammals.

[33] Dudhat S., Pande A., Nair A., Mondal I., Srinivasan M., Sivakumar K. (2002). Spatio-temporal analysis identifies marine mammal stranding hotspots along the Indian coastline. Sci. Rep..

[34] Gulland F.M.D., Dierauf L.A., Whitman K.L. (2018). CRC Handbook of Marine Mammal Medicine.

[35] NOAA Fisheries Marine Mammal Unusual Mortality Events. https://www.fisheries.noaa.gov/national/marine-mammal-protection/marine-mammal-unusual-mortality-events.

[36] NOAA Fisheries 2013–2015 Bottlenose Dolphin Unusual Mortality Event in the Mid-Atlantic (Closed). https://www.fisheries.noaa.gov/national/marine-life-distress/2013-2015-bottlenose-dolphin-unusual-mortality-event-mid-Atlantic.

[37] Stephens N., Duignan P.J., Wang J., Bingham J., Finn H., Bejder L., Patterson A.P., Holyoake C. (2014). Cetacean morbillivirus in coastal Indo-Pacific bottlenose dolphins, Western Australia. Emerg. Infect. Dis..

[38] Van Bressem M.F., Duignan P.J., Banyard A., Barbieri M., Colegrove K.M., De Guise S., Di Guardo G., Dobson A., Domingo M., Fauquier D. (2014). Cetacean morbillivirus: Current knowledge and future directions. Viruses.

[39] Pyati R., Bielmyer G.K., Chalk S., McCarthy D., McCarthy H., Pinto G., Sonnenberg L., Welsh P. (2012). Case Study: St. Johns River Basin, USA.

[40] Environmental Protection Agency, United States (1998). Method 7473 (SW-846): Mercury in Solids and Solutions by Thermal Decomposition, Amalgamation, and Atomic Absorption Spectrophotometry. Revision 0. https://www.epa.gov/esam/epa-method-7473-sw-846-mercury-solids-and-solutions-thermal-decomposition-amalgamation-and.

[41] Bellante A., Sprovieri M., Buscaino G., Buffa G., Di Stefano V., Salvagio Manta D., Barra M., Filiciotto F., Bonanno A., Giacoma C. (2012). Stranded cetaceans as indicators of mercury pollution in the Mediterranean Sea. Ital. J. Zool..

[42] Cardellicchio N., Decataldo A., Di L.A., Misino A. (2002). Accumulation and tissue distribution of mercury and selenium in striped dolphins (*Stenella coeruleoalba*) from the Mediterranean Sea (southern Italy). Environ. Pollut..

[43] García-Alvarez N., Fernández A., Boada L.D., Zumbado M., Zaccaroni A., Arbelo M., Sierra E., Almunia J., Luzardo O.P. (2015). Mercury and selenium status of bottlenose dolphins (*Tursiops truncatus*): A study in stranded animals on the Canary Islands. Sci. Total Environ..

[44] Squadrone S., Chiaravalle E., Gavinelli S., Monaco G., Rizzi M., Abete M.C. (2015). Analysis of mercury and methylmercury concentrations, and selenium: Mercury molar ratios for a toxicological assessment of sperm whales (*Physeter macrocephalus*) in the most recent stranding event along the Adriatic coast (Southern Italy, Mediterranean Sea). Chemosphere.

[45] Caceres-Saez I., Haro D., Blank O., Aguayo Lobo A., Dougnac C., Arredondo C., Cappozzo H.L., Ribeiro Guevara S. (2018). High status of mercury and selenium in false killer whales (*Pseudorca crassidens*, Osen 1846) stranded on Southern South America: A possible toxicological concern. Chemosphere.

[46] Moreira I., Seixas T.G., Kehrig H.A., Fillmann G., Di Beneditto A.P., Souza C.M., Malm O. (2009). Selenium and mercury (total and organic) in tissues of a coastal small cetacean, *Pontoporia blainvillei*. J. Coast. Res..

[47] Sedak M., Bilandžić N., Đokić M., Đuras M., Gomerčić T., Benić M. (2002). Body burdens and distribution of mercury and selenium in bottlenose, striped and Risso’s dolphins along the Adriatic coast: A 20-year retrospective. Mar. Pollut. Bull..

[48] Endo T., Kimura O., Hisamichi Y., Minoshima Y., Haraguchi K., Kakumoto C., Kobayashi M. (2006). Distribution of total mercury, methyl mercury and selenium in pod of killer whales (*Orcinus Orca*) stranded in the northern area of Japan: Comparison of mature females with calves. Environ. Pollut..

[49] Hansen A.M., Bryan C.E., West K., Jensen B.A. (2016). Trace element concentrations in liver of 16 species of cetaceans stranded on Pacific Islands from 1997 through 2013. Arch. Environ. Contam. Toxicol..

[50] Kershaw J.L., Hall A.J. (2019). Mercury in cetaceans: Exposure, bioaccumulation and toxicity. Sci. Total Environ..

[51] Roditi-Elasar M., Kerem D., Hornung H., Kress N., Shoham-Frider E., Goffman O., Spanier E. (2003). Heavy metal levels in bottlenose and striped dolphins off the Mediterranean coast of Israel. Mar. Pollut. Bull..

[52] Martínez-López E., Peñalver J., Lara L., Garcia-Fernandez A.J. (2019). Hg and Se in Organs of Three Cetacean Species from the Murcia Coastline (Mediterranean Sea). Bull. Environ. Contam. Toxicol..

[53] Wagemann R., Trebacz E., Boila G., Lockhart W.L. (1998). Methylmercury and total mercury in tissues of arctic marine mammals. Sci. Total Environ..

[54] Rawson A.J., Patton G.W., Hofmann S., Pietra G.G., Johns L. (1993). Liver Abnormalities Associated with Chronic Mercury Accumulation in Stranded Atlantic Bottlenosed Dolphins. Ecotoxicol. Environ. Saf..

[55] Meador J.P., Ernest D., Hohn A.A., Tilbury K., Gorzelany J., Worthy G., Stein J.E. (1999). Comparison of elements in bottlenose dolphins stranded on the beaches of Texas and Florida in the Gulf of Mexico over a one-year period. Arch. Environ. Contam. Toxicol..

[56] Ralston N.V.C., Azenkeng A., Raymond L.J., Ceccatelli S., Aschner M. (2012). Mercury-Dependent Inhibition of Selenoenzymes and Mercury Toxicity. Methylmercury and Neurotoxicity. Current Topics in Neurotoxicity.

[57] Nakazawa E., Ikemoto T., Hokura A., Terada Y., Kunito T., Tanabe S., Nakai I. (2011). The presence of mercury selenide in various tissues of the striped dolphin: Evidence from μ-XRF-XRD and XAFS analyses. Metallomics.

[58] Marumoto M., Sakamoto M., Nakamura M., Marumoto K., Tsuruta S. (2022). Organ-specific accumulation of selenium and mercury in Indo-Pacific bottlenose dolphins (*Tursiops aduncus*). Acta. Vet. Scand..

[59] Pinto G., Bielmyer-Fraser G.K., Baynard C.D., Closmann C., Goldberg N., Jones S., Johnson A., Penwell W., Pyati R., Rosenblatt A. (2023). State of the River Report for the Lower St. Johns River Basin, Florida: Water Quality, Fisheries, Aquatic Life, & Contaminants.

[60] Marine Mammal Health and Stranding Response Program. https://www.fisheries.noaa.gov/national/marine-life-distress/marine-mammal-health-and-stranding-response-program.

